# Unveiling the Microstructure Evolution and Mechanical Strengthening Mechanisms in Mg–2Y–*x*Zn Alloys

**DOI:** 10.3390/ma18143303

**Published:** 2025-07-14

**Authors:** Luyan Xu, Huanjian Xie, Kuan Chen, Ruizhi Feng, Donghui Zheng, Haoge Shou

**Affiliations:** 1 School of Intelligent Manufacturing, Huanghuai University, Zhumadian 463000, China; luyanxu163@163.com (L.X.); zhengdonghui@huanghuai.edu.cn (D.Z.); shouhaoge@huanghuai.edu.cn (H.S.); 2 School of Materials Science and Engineering, Dalian University of Technology, Dalian 116024, China; 3 Zhumadian City Key Laboratory of High-Performance Magnesium Alloy Research and Development, Huanghuai University, Zhumadian 463000, China; 4 China Railway Rolling Stock Corporation Qishuyan Locomotive and Rolling Stock Technology Research Institute Co., Ltd., Changzhou 213000, China; broadchen97@gmail.com; 5 College of Automation Engineering, Nanjing University of Aeronautics and Astronautics, Nanjing 210016, China; xy199908282519@163.com

**Keywords:** Mg–2Y–*x*Zn alloys, phase evolution, mechanical strengthening mechanism, synergistic strengthening

## Abstract

This work systematically investigates the Zn-content-dependent phase evolution (1–12 at.%) and its correlation with mechanical properties in as-cast Mg–2Y–*x*Zn alloys. A sequential phase transformation is observed with the Zn content increasing: the microstructure evolves from X-phase dominance (1–2 at.% Zn) through W-phase formation (3–6 at.% Zn) to I-phase emergence (12 at.% Zn). Optimal mechanical performance is attained in the 2 at.% Zn-containing alloy, with measured tensile properties reaching 239 MPa UTS and 130 MPa YS, while maintaining an elongation of 12.62% prior to its gradual decline at higher Zn concentrations. Crystallographic analysis shows that the most significant strengthening effect of the X-phase originates from its coherent orientation relationship with the α-Mg matrix and the development of deformation-induced kink bands. Meanwhile, fine W-phase particles embedded within the X-phase further enhance alloy performance by suppressing X-phase deformation, revealing pronounced synergistic strengthening between the two phases. Notably, although both the I-phase and W-phase act as crack initiation sites during deformation, their coexistence triggers a competitive fracture mechanism: the I-phase preferentially fractures to preserve the structural integrity of the W-phase, effectively mitigating crack propagation. These dynamic interactions of second phases during plastic deformation—synergistic strengthening and competitive fracture—provide a novel strategy and insights for designing high-performance Mg–RE–Zn alloys.

## 1. Introduction

Magnesium (Mg), the lightest structural metal, holds immense application potential across industries due to its exceptional specific strength and specific stiffness, making it an ideal candidate for lightweight design in automotive, aerospace, and biomedical sectors [1,2,3]. However, its practical utilization is often constrained by relatively low mechanical strength and poor ductility [4]. Alloying has emerged as a pivotal strategy to overcome these limitations, thereby improving the industrial suitability of Mg alloys [5].

Mg–Y–Zn alloys stand out as a paradigm due to their unique microstructural characteristics and remarkable mechanical improvements in various alloy systems [6,7]. For instance, the Mg_97_Y_2_Zn_1_ alloy, fabricated using rapid solidification powder metallurgy, demonstrates an elongation of 5% and a yield strength of 610 MPa at 25 °C. The enhanced mechanical performance primarily stems from the X-phase (Mg_12_YZn) containing nanoscale long-period stacking ordered (LPSO) structures, which effectively suppress twin propagation during deformation [8]. Extensive studies demonstrate that yttrium (Y) and zinc (Zn) additions promote the formation of distinct intermetallic phases—X-phase, W-phase (Mg_3_Zn_3_Y_2_), and I-phase (Mg_3_Zn_6_Y)—each playing critical roles in mechanical strengthening [9,10,11]. For the X-phase, its unique LPSO structure exhibits coherent interfaces with the Mg matrix along both basal and prismatic planes, as evidenced by HRTEM observations [12]. This strong interfacial bonding suppresses microcrack initiation and propagation at LPSO/Mg interfaces under stress [12,13,14]. Furthermore, kink deformation behavior, a distinctive feature of LPSO structures in Mg alloys, serves as a vital mechanism for homogeneous strain accommodation, contributing synergistically to both strength and ductility [15,16]. The W-phase also constitutes a key component in Mg–Y–Zn alloys. Studies reveal that nano-sized W particles enhance the strength and ductility of Mg–Zn–Y alloys [17,18,19]. However, coarse W particles may deteriorate strength due to limited symmetry and incoherent interfacial relationships between W-phase and α-Mg matrix [18,20]. Thus, the role of the W-phase predominantly depends on precise control of particle size and distribution [21]. The I-phase, characterized by an icosahedral quasicrystal structure, exhibits high Young’s modulus but inherent brittleness [22]. Surprisingly, coarse I-phase particles with an icosahedral quasicrystal structure tend to fracture, which weakens the load transfer from the α-Mg matrix to the I-phase in the course of plastic deformation [20,23]. In contrast, nano-sized I-phase particles interact effectively with dislocations within parent grains and twins [24]. Although the individual contributions of these phases to mechanical enhancement are well documented, limited attention has been paid to their dynamic interactions during deformation or their collective influence on overall performance. Understanding these interphase interactions is an important concern for optimizing the design and functionality of Mg–RE–Zn alloys.

This work systematically investigates the alloy composition, intermetallic phase formation, crystallographic orientation relationships, mechanical properties and mechanisms within the Mg–2Y–*x*Zn Alloys. A series of the alloys (*x* = 1, 2, 3, 6, and 12 at.%, designated as WZ21, WZ22, WZ23, WZ26, and WZ212, respectively) were designed to explore microstructure evolution and mechanical responses, aiming to uncover novel mechanisms for mechanical performance enhancement.

## 2. Experimental Methods

The investigation focused on five Mg–2Y–*x*Zn (atomic percent) alloys. These alloys were individually synthesized using high-purity Mg (99.9%), a Mg-30Y (weight percent) precursor alloy, and pure Zn (99.98%). The alloy melting was carried out in a resistance furnace under precise thermal regulation (720 ± 5 °C) within a CO_2_-SF_6_ mixed shielding environment. Following the melting process, the liquid alloys were cast into an iron mold measuring 50 mm × 100 mm × 150 mm. The variable *x* assumed the values of 1, 2, 3, 6, and 12, leading to the creation of the Mg–2Y–*x*Zn alloys which were correspondingly labeled as WZ21, WZ22, WZ23, WZ26, and WZ212. The actual compositions of the investigated alloys were measured by an inductively coupled plasma optical spectrometry (ICP-6300) and are given in Table 1 (Mg–Y–Zn alloys are referred as WZ).

Tensile tests were performed at room temperature using a WDW-10S tensile testing machine (Shandong, China) at a constant cross-head speed of 0.45 mm/min. Specimens with a rectangular cross-section (2 mm thickness × 3.5 mm width) and a 14 mm gauge length were machined from the cast plates. At least three specimens were tested for each alloy composition under identical conditions. YS was determined at 0.2% plastic strain offset, UTS as the maximum stress, and EL as fracture strain. We have made the appropriate revisions in the manuscript. To accurately determine the true phase composition of the as-cast alloys, X-ray diffraction (XRD, Rigaku SmartLab SE, Tokyo, Japan) was employed for analysis. The tests utilized a Cu Kα radiation source, with an operating voltage set to 40 kV, a scanning rate of 5°/min, and 2θ angle range from 10° to 90°. Then, Jade software (Jade 6.5) was used to analyze the data. The microstructures and intermetallic compound of these alloys were characterized using a Sigma 300 scanning electron microscope (SEM) (St. Louis, MO, USA) integrated with an Oxford Xplore 50 energy-dispersive X-ray spectroscopy (EDS) system (Oxford, UK). To delve deeper into the alloy’s refined microstructure, a Jeol 2100F field emission TEM (Tokyo, Japan) operating at 200 kV was utilized, along with SAED.

## 3. Results and Discussion

### 3.1. Phase Composition

**Figure 1:**
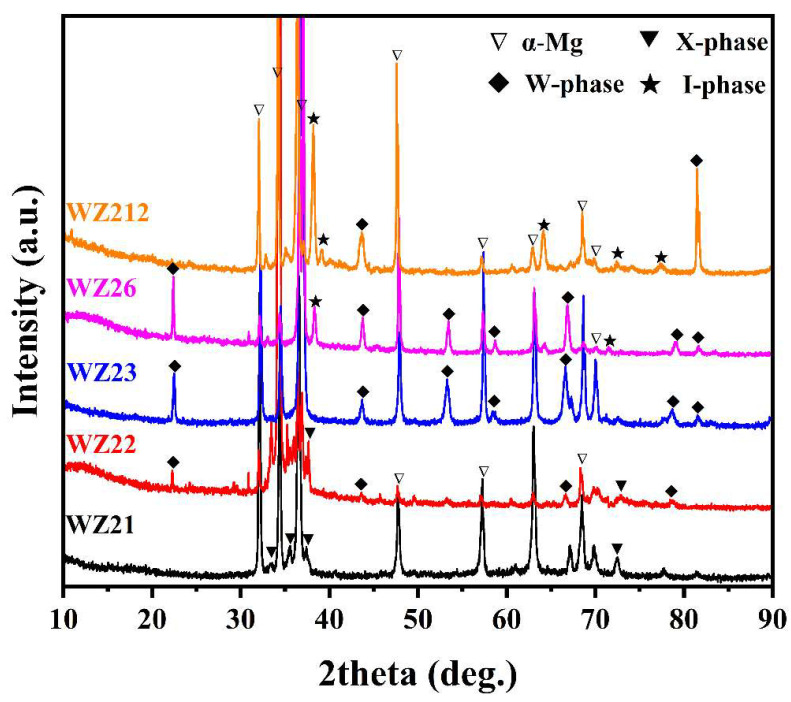
X-ray diffraction analysis results of the as-cast Mg–2Y–*x*Zn alloys.

Figure 1 displays the XRD patterns of as-cast Mg–2Y–*x*Zn alloys across varying Zn compositions. Alongside the α-Mg matrix, the predominant second phases detected in the five alloys are identified as X-phase, W-phase, and I-phase, with phase assignments validated via ICDD PDF card 2004. Based on the XRD patterns, the WZ21 alloy primarily contains the X-phase, while the WZ22 alloy comprises both the X- and W-phases due to its higher Zn content. When the Zn content increases to 3 at.%, the WZ23 alloy consists exclusively of the W-phase. When the content of Zn exceeds 3 at.%, the I-phase starts to appear and also contains the W-phase, the two alloys are WZ26 and WZ212 respectively. It is evident that the phase composition in the Mg–2Y–*x*Zn alloy system varies in response to changes in the Zn content. When the content of Zn increases, the second phase is more inclined towards the second phase with a larger Zn/Y ratio. Therefore, the predominant second phases in Mg–2Y–*x*Zn alloys systematically transition between X-phase, W-phase, and I-phase with progressive Zn content variation.

### 3.2. Microstructure Formation Process

**Figure 2:**
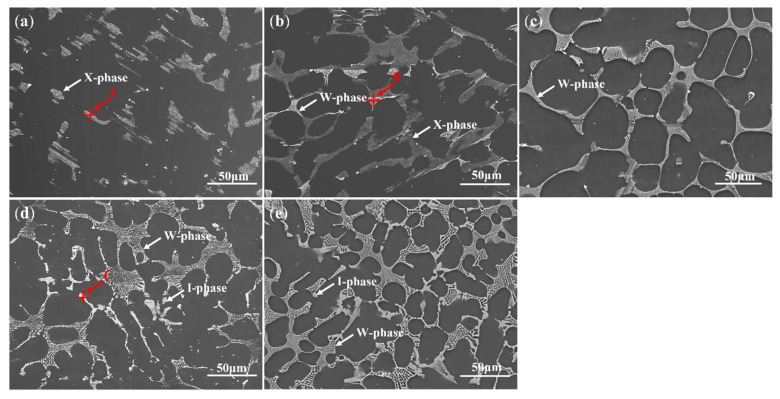
SEM images depicting the Mg–2Y–*x*Zn alloys with increasing Zn content: (**a**) WZ21, (**b**) WZ22, (**c**) WZ23, (**d**) WZ26, (**e**) WZ212.

Figure 2 indicates SEM images of Mg–2Y–*x*Zn alloys, illustrating the distribution of phases. From the figure, it is apparent that the alloy series primarily consists of a gray matrix and white particles. In addition, it contains gray lath-shaped, reticular-shaped, and block-shaped second phases. When the content of Zn is 1 at.%, the alloy mainly contains white particles and gray lath-shaped intermetallic phase. When the percentage of Zn is increased to 2 at.%, the alloy contains mainly grey laths and white reticulated eutectic phases. When the amount of Zn rises to 3 at.%, the alloy has only a reticular eutectic phase. When the Zn reaches 6 at.% and 12 at.%, in addition to the reticulated eutectic phase, the block second phase also starts to appear in the alloy and the number of reticulated eutectic phases decreases. The amount of block second phase rises significantly and the total scalar of phases increases at a Zn content of 12 at.% compared to a Zn content of 6 at.%. The comparison shows that when the Zn percentage is below 3 at.%, the reticular eutectic phase gradually grows with the increase of Zn content, and the white particles and lath-shaped second phase gradually decrease until they disappear. When the Zn content reaches 6 at.%, the block second phase starts to appear in the alloy, and as the Zn content increases, the reticular eutectic phase gradually decreases and the block second phase gradually increases. EDS analysis was conducted to characterize the chemical components of the second phases.

**Figure 3:**
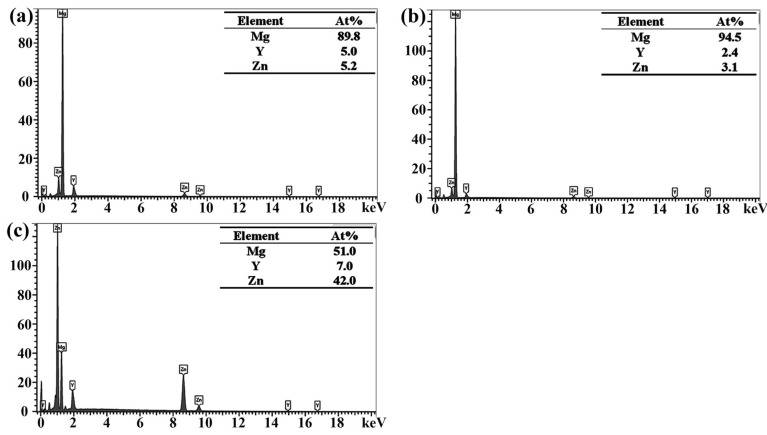
The EDS results of the main second phases: (**a**) X-phase, (**b**) W-phase, (**c**) I-phase, corresponding to points A, B, and C marked in Figure 2, respectively.

The dominant second phases were analyzed by EDS (Figure 3), with subfigures (a)–(c) corresponding to points A, B, and C marked in Figure 2, respectively. The ratios of Y/Zn in the gray lath-shaped, reticular-shaped, and block-shaped secondary phases are approximately 1:1, 2:3, and 1:6, respectively, as indicated by the results. The analysis of the XRD findings (Figure 1) combined with the microstructural features (Figure 2) enables the identification of these phases. The lath-shaped microstructure is identified as the X-phase, while the reticular eutectic structure corresponds to the W-phase, and the bulk morphology is attributed to the I-phase. Additionally, the formation of the W-phase is promoted when the Zn content is increased up to 3 at.%. The I-phase starts to form at 6 at.% Zn content and its formation increases progressively with further increases in Zn content. In the alloy series, the X-phase is preferentially generated for the WZ21 alloy because the ratio of Zn/Y is 1. Additionally, the tiny white particles are identified as Mg_24_Y_5_ and their generation is attributed to Y elemental segregation in the process of microstructure formation. Stanford et al. [25] have studied the grain boundary segregation of rare-earth elements in magnesium alloys. Combined with the SEM image, we can observe that the white particles are mostly produced at the grain boundaries. As for the WZ23 alloy, the W-phase is exclusively observed at a Zn content of 3 at.%. In contrast, the WZ22 system exhibits dual-phase characteristics (X-phase and W-phase) within the 1–3 at.% Zn compositional range. Progressive Zn additions (6–12 at.%) elevate the Zn/Y atomic ratio beyond 1.5, inducing preferential formation of zinc-enriched intermetallic compounds (I-phase) during solidification. This phase evolution mechanism corroborates findings reported in analogous Mg–RE–Zn ternary systems by Lee et al. [26] and Zhu et al. [27].

Based on the literature [28,29], the transformation sequence of the four main phases as the Mg–2Y–*x*Zn alloy solidifies can be obtained. The phase transition sequences during solidification of Mg–2Y–*x*Zn alloy systems are summarized in Table 2. The WZ22 alloy comprises the X-phase, W-phase, and α-Mg. The alloy solidifies first with α-Mg precipitating from the liquid phase at 620 °C:

$$L\;\overset{620\;{}^{\circ}C}{\to }\alpha ‑Mg+L$$

The eutectic melting points of X-phase (Mg_12_ZnY) and Mg_24_Y_5_ are 530° C, whereas that of W-Mg_3_Y_2_Zn_3_ is 528 °C. As a result, the WZ22 alloy undergoes a ternary eutectic reaction at 528 °C:

$$L\;\overset{528\;{}^{\circ}C}{\to }{Mg}_{12}ZnY+{Mg}_{24}Y_{5}+{Mg}_{3}Y_{2}{Zn}_{3}$$

The WZ26 alloy contains both the I-phase and the W-phase. Despite its unique morphology and varied growth orientation, the I-phase forms via the classical nucleation and growth mechanism. As evidenced by prior studies [30], a eutectic reaction is predominantly triggered upon cooling the melt to 550 °C as follows:

$$L\;\overset{550\;{}^{\circ}C}{\to }\alpha ‑Mg+{Mg}_{3}Y_{2}{Zn}_{3}$$

Here, Mg_3_Zn_3_Y_2_ is called the W-phase, and Mg_3_YZn_6_ corresponds to the I-phase. As the melt temperature decreases, the I-phase/α-Mg eutectic pocket forms via peritectic reaction at 448 °C:

$$L+W\overset{448\;{}^{\circ}C}{\to }\alpha ‑Mg+{Mg}_{3}Y{Zn}_{6}$$

**Figure 4:**
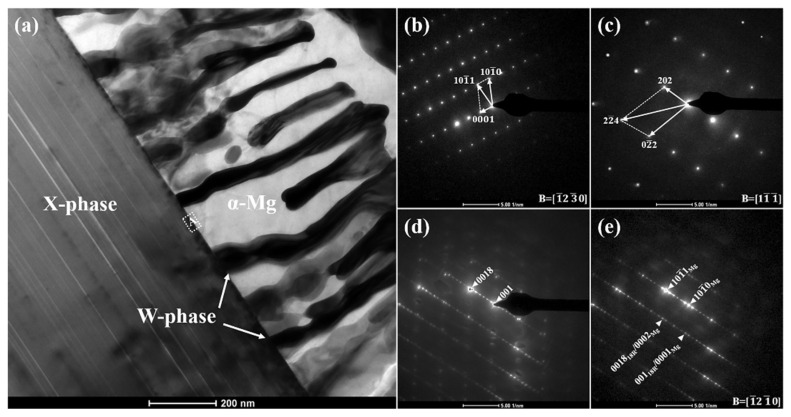
Microstructural characterization of WZ22 alloy: (**a**) TEM image showing phase distribution and interfacial regions; (**b**–**d**) SAED patterns corresponding to (**b**) α-Mg matrix, (**c**) W-phase, and (**d**) X-phase, indexed along the zone axes B = [$\bar{1}$230] and B = [1$\bar{1}\bar{1}$], respectively; (**e**) Composite diffraction pattern acquired from the α-Mg/X-phase interface (marked as region A in (**a**)), demonstrating crystallographic orientation relationships between the two phases.

Figure 4 displays the TEM micrograph and associated SAED patterns of the as-cast WZ22 alloy. The alloy comprises three distinct phases, as illustrated in Figure 4a. The black striated phases are alternately and uniformly distributed within the white massive matrix phase, with a clear phase interface between them. According to the SAED patterns shown in Figure 4b,c, the matrix is identified as α-Mg, while the striated phase corresponds to the W-phase (*a* = 0.6848 nm, space group Fm3m) [31]. The partial ordering of the AlMnCu_2_-type compound in the W-phase results in an incoherent interface with α-Mg and limited lattice axes, leading to weakened atomic bonding [32]. Figure 4d displays the SAED pattern of a layered phase with five additional diffraction spots located at n/6(0002)_α-Mg_ positions (where n represents the order of the diffraction spots), confirming the presence of an 18R long-period stacking ordered (LPSO) structure [9,10,11,12]. Furthermore, the composite diffraction pattern in Figure 4e, obtained from the interfacial region labeled A in Figure 4a, illustrates the crystallographic orientation relationship between the X-phase and the α-Mg matrix. The X-phase aligns closely with the [1210] zone axis of α-Mg, following the orientation relationship (0001)_α-Mg_//(001)_18R_ and [1210]_α-Mg_//[010]_18R_. The symmetric lattice of the X-phase contributes to strong atomic bonding with the matrix. Additionally, previous studies indicate that the (0111)_18R_ plane of the X-phase is parallel to the (002)_W_ plane of the W-phase, with a lattice mismatch of ~33% [33]. This significant mismatch further confirms the incoherent nature of the interface between the X-phase and W-phase. The orientation between the W-phase and α-Mg can be represented as [001]_W_//[01$\bar{1}$0]_α-Mg_, (110)_W_//(0001)_α-Mg_ [34].

**Figure 5:**
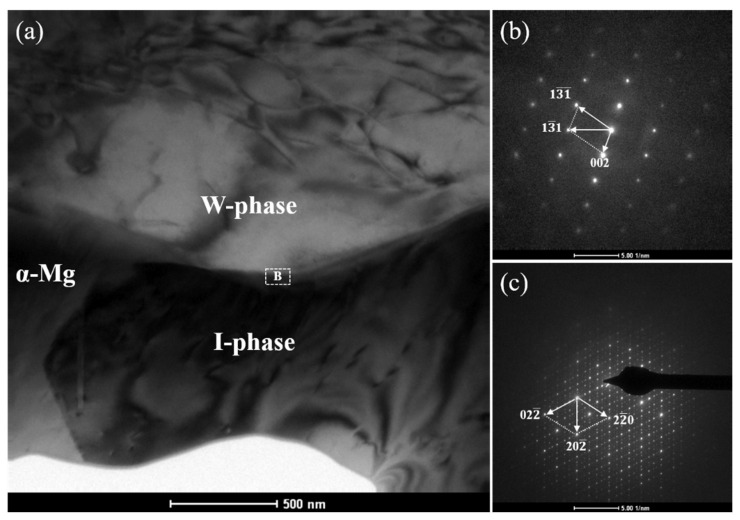
TEM characterization of WZ26 alloy: (**a**) Bright-field image showing characteristic microstructure; (**b**) SAED pattern indexing to the W-phase, (**c**) composite diffraction from zone B (marked in (**a**)) revealing W-phase/I-phase interface relationships.

Figure 5 displays the TEM micrograph and corresponding SAED patterns of the as-cast WZ26 alloy. Combined with the XRD results (Figure 1) and SEM analysis (Figure 2d), the TEM image in Figure 5a reveals three distinct phases in the alloy. Based on the SAED patterns in Figure 5b,c, the hourglass-shaped phase is identified as the icosahedral quasicrystal phase (I-phase), while the light-colored bulky phase corresponds to the W-phase (space group Fm3m). As shown in Figure 5c, the SAED pattern of the I-phase reveals a two-fold rotational symmetry, a characteristic feature of quasicrystals, consistent with previous reports by Luo and Tang [29,30]. Notably, the interface between the I-phase and W-phase is not atomically sharp but exhibits a (quasi-)epitaxially coherent character. This coherence suggests strong atomic bonding at the I/W interfaces. From a crystallographic perspective, the two phases exhibit a high degree of lattice matching. Research conducted by Singh and Tsai [34] indicates that the icosahedral quasicrystal is capable of forming low-energy interfaces with crystalline phases like the W-phase, owing to its high degree of rotational symmetry. Specifically, the quasi-periodic nature of the I-phase allows variable interplanar spacing, which enhances atomic registry at the interface. These observations collectively indicate a strong site-specific orientation relationship between the W-phase and I-phase. Additionally, the I/α-Mg interfaces exhibit low interfacial energy, which is attributed to their structural compatibility and minimal lattice distortion. This characteristic contributes to high resistance to interfacial cracking during mechanical loading, as documented in prior research on Mg-based alloys [20].

**Figure 6:**
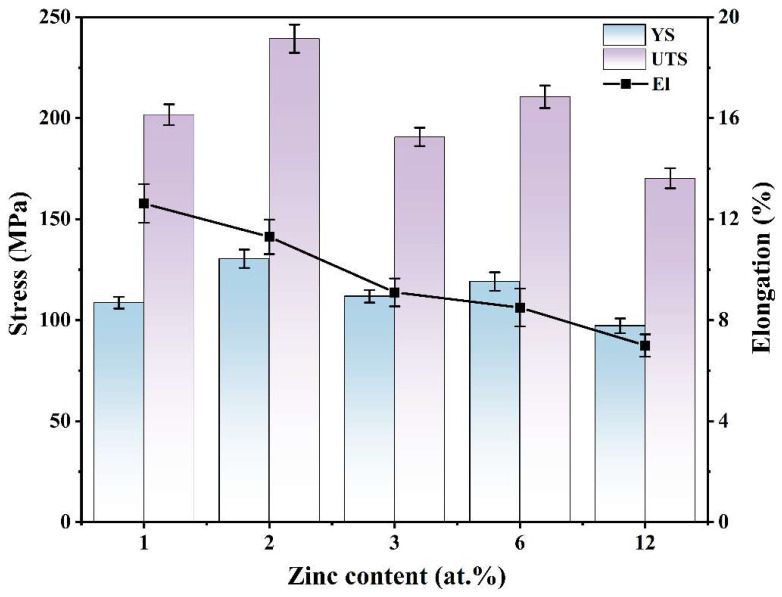
Mechanical properties of as-cast Mg–2Y–*x*Zn alloys. (YS was determined at 0.2% plastic strain offset, UTS as the maximum stress, and EL as fracture strain).

Figure 6 illustrates how the mechanical properties of as-cast Mg–2Y–*x*Zn alloys are influenced by varying zinc content, with a focus on UTS, YS, and EL. Figure 6 delineates the mechanical behavior of as-cast Mg–2Y–*x*Zn alloys, emphasizing the role of Zn content in modulating UTS, YS, and EL. Both UTS and YS exhibit synchronous variation trends with zinc content adjustments. At Zn levels ≤ 3 wt%, both parameters initially escalate to a maximum before decreasing. Beyond 3 wt% Zn, a comparable pattern is observed, wherein UTS and YS rise transiently before diminishing. A progressive decline in EL with rising Zn concentration underscores the inverse correlation between Zn addition and ductility. The WZ22 alloy (2 wt% Zn) attains peak values of 239 MPa (UTS) and 130 MPa (YS), marking the highest strength in the system. In contrast, the WZ21 alloy (1 wt% Zn) exhibits superior ductility, achieving an EL of 12.62%, the highest among all compositions.

In general, the mechanical performance of alloys is strongly affected by the morphology, dispersion, dimensions, and nature of intermetallic phases. Figure 2 depicts the features of intermetallic compounds within the investigated alloys. The X-phase is the dominant intermetallic compound in the WZ21 alloy. The WZ22 alloy contains two main phases, X- and W-phases, while only the W-phase exists in the WZ23 alloy. As the Zn content increases, both the WZ26 and WZ212 alloys form the I-phase, and these alloys contain both the I- and W-phases. The X-phase, owing to its LPSO structure, is recognized by earlier research as the most effective strengthening phase in magnesium alloys [35]. In contrast, the W-phase, which is large and characterized by its hardness and brittleness, is susceptible to fracture during tensile deformation, leading to a decline in mechanical properties [18,20]. Consequently, the presence of the X-phase ensures favorable mechanical properties for the WZ21 alloy, whereas the W-phase leads to unsatisfactory mechanical performance in the WZ23 alloy. Interestingly, however, relative to the WZ21 alloy, the presence of the W-phase in the WZ22 alloy does not negatively affect its mechanical properties. On the contrary, UTS and YS increase, which is contrary to conventional theoretical predictions. Significantly, the I-phase demonstrates coherent interfaces with both the Mg matrix and the W-phase, confirming its established role as a potent strengthening constituent in magnesium alloys [24,34]. The enhanced mechanical performance of the WZ26 alloy over WZ23 is likely because of the existence of the I-phase. Paradoxically, although the WZ212 alloy contains elevated I-phase content, its mechanical characteristics show marked degradation relative to WZ26. Thus, the mechanical strengthening mechanisms of the WZ22 and WZ212 alloys cannot be adequately explained by single-phase strengthening theories. However, their phase compositions (X + W for WZ22 and W + I for WZ212) suggest the potential existence of unique dynamic interphase interactions during plastic deformation, which may represent a novel strengthening mechanism in magnesium alloys. To clarify this hypothesis, further analysis was conducted on post-deformation samples.

**Figure 7:**
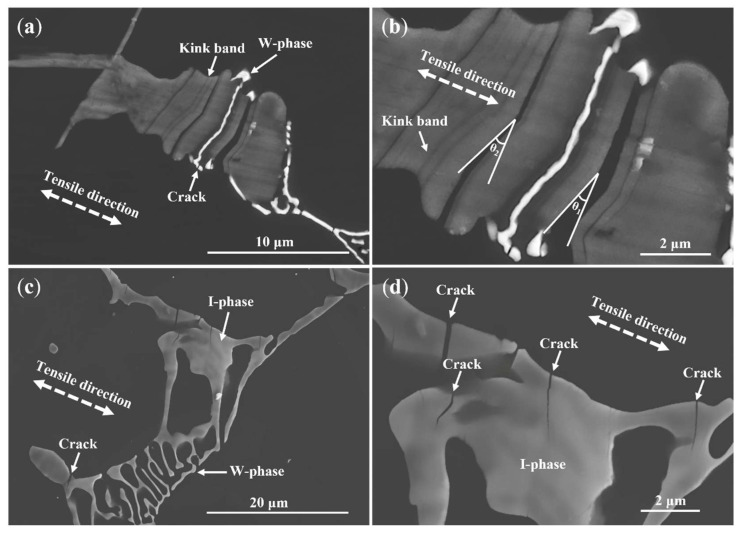
SEM images of the longitudinal sections near the fracture surfaces of the alloys after tensile testing: (**a**,**b**) WZ22, (**c**,**d**) WZ26.

Figure 7 displays SEM images of the longitudinal sections of WZ22 and WZ26 alloys following tensile deformation. For the WZ22 alloy, the formation of kink bands within the X-phase is evident during deformation (Figure 7a,b), consistent with previous studies by Shao et al. [36], who identified kink banding as a predominant deformation feature in X-phase-dominated microstructures. Xu et al. [29] further emphasized that kinking is critical for achieving uniform strain distribution and enhancing ductility [15,16]. Notably, microcracks were observed at the interfaces between the X-phase and the nanolamellar Mg-rich interlayers during later deformation stages (Figure 7b). However, these cracks propagated solely within individual kink bands, thereby suppressing premature fracture [36]. Crystallographic analysis (Figure 5) confirmed a coherent orientation relationship between the X-phase and the Mg matrix: (0001)Mg/(001)18R and [1210]Mg/[010]_18R_, which accounts for the absence of interfacial cracking and highlights the robust bonding between the X-phase and Mg matrix. These results collectively demonstrate that the kink-induced deformation mechanism, coupled with the favorable crystallographic alignment, underpins the X-phase strengthening effect.

Intriguingly, the W-phase embedded within the X-phase remained intact with minimal deformation (Figure 7a,b), suggesting its role in impeding X-phase kinking. Quantitative analysis revealed distinct kink angles in regions adjacent to the W-phase (θ_1_ = 20°) compared to regions without W-phase obstruction (θ_2_ = 23°). The ~15% increase in θ_2_ underscores the pronounced inhibitory effect of the W-phase on X-phase deformation, confirming a synergistic strengthening mechanism between the two phases. In contrast, the W-phase within the Mg matrix of WZ22 fractured extensively (Figure 7a), generating microcracks that propagated during deformation, ultimately leading to alloy failure. This disparity arises from the inherent hard-brittle nature of the W-phase, which exacerbates crack initiation in the Mg matrix but enhances load transfer efficiency when embedded within the X-phase.

For the WZ26 alloy (Figure 7c,d), the I-phase exhibited preferential fracture with cracks propagating perpendicular to the tensile direction, attributed to its low stacking fault energy and quasi-crystalline structure [24]. Remarkably, cracks nucleated exclusively within the I-phase rather than at the W/I interfaces (Figure 5), corroborating the strong interfacial cohesion between these phases. Despite sharing similar hard-brittle characteristics, the W-phase remained undamaged, while the I-phase underwent catastrophic failure. This competitive fracture behavior implies a phase-dependent stress redistribution mechanism, wherein the I-phase acts as a sacrificial component, shielding the W-phase and preserving its structural integrity during deformation. Such a competitive interaction between the W- and I-phases provides a novel pathway for optimizing the trade-off between strength and ductility in Mg–RE–Zn alloys.

**Figure 8:**
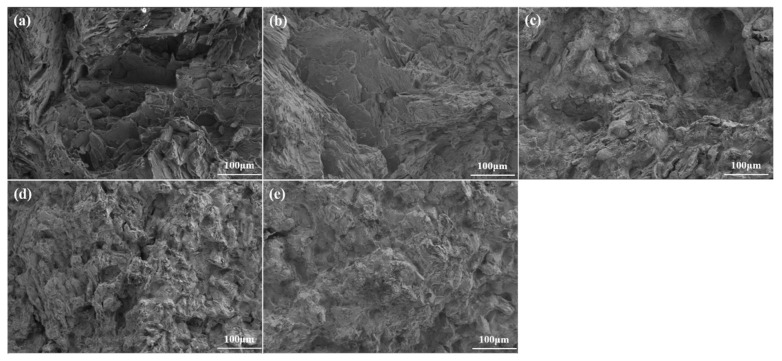
SEM micrographs of tensile-induced fracture patterns: (**a**) WZ21, (**b**) WZ22, (**c**) WZ23, (**d**) WZ26, and (**e**) WZ212.

Figure 8 presents the fracture morphologies of as-cast Mg–2Y–*x*Zn alloys under tensile loading, which were examined using a SEM. The fracture behavior exhibits a significant dependence on the alloy composition. For the WZ21 alloy (Figure 8a), the fracture surface is predominantly characterized by large and deep plastic dimples and tear ridges, indicating a ductile fracture mechanism. In contrast, as the Zn content increases in the WZ22, WZ23, WZ26, and WZ212 alloys (Figure 8b–e), they display mixed quasi-cleavage fracture characteristics. In these alloys, cleavage planes and fragmented W- and I-phase particles are quite evident, suggesting a transition in the fracture mode. Fractographic analysis of the WZ22, WZ23, WZ26, and WZ212 alloys (Figure 8b–e) show the coexistence of transgranular dimple cracking and intergranular cleavage, and fragmented second-phase particles are embedded in the dimples.

These observations are consistent with the strengthening and failure mechanisms of the second phases: Combining Figure 2, Figure 4 and Figure 7a,b, for the WZ21 and WZ22 alloys, the X-phase has a good orientation relationship ((001)_18R_//(0001)_α-Mg_ and [010]_18R_//[1210]_α-Mg_) with the magnesium matrix. During the plastic deformation process, its interface is not prone to cracking, which could lead to alloy failure. Additionally, kink bands form within the X phase due to the occurrence of plastic deformation. This unique strengthening mechanism significantly improves the alloy’s mechanical performance. Conversely, the body-centered cubic W-phase and quasi-crystalline icosahedral I-phase (dominant in the WZ23, WZ26, and WZ212 alloys) exhibit inherent brittleness. During plastic deformation, stress concentration causes microcrack nucleation and premature failure. This explains the decrease in the elongation of the alloys containing the W- and I-phases, which is consistent with previous research results [18,20,21,22,23,24]. Notably, combining Figure 7a,b, there is a synergistic strengthening mechanism between the X-phase and the W-phase, contributing to the enhancement of the alloy’s mechanical properties to some degree. Notwithstanding this, the ultimate failure mechanism of the alloy remains attributed to the initiation and coalescence of micro-cracks at the W-phase under tensile stress concentration. Therefore, obvious fragmented W-phases can also be identified at the fracture site of the WZ22 alloy. For the WZ26 and WZ212 alloys, the I-phase acts as a sacrificial component and becomes the location for crack initiation, protecting the W-phase and maintaining its structural integrity during deformation. In summary, the X-phase significantly plays a crucial role in enhancing the alloy’s strength, while the W- and I-phases become the locations for crack initiation. This highlights the importance of selecting appropriate phases when balancing the alloy’s performance under mechanical stress.

## 4. Conclusions

1. The Mg–2Y–*x*Zn alloy system exhibits a dynamic evolution of phase composition with increasing Zn content. Initially, the alloy containing 1 at.% Zn is dominated by the X-phase. As Zn content rises to 3 at.%, the X-phase progressively diminishes and ultimately disappears, while the W-phase emerges and intensifies. Beyond 3 at.% Zn, the I-phase forms and steadily increases up to 12 at.% Zn.
2. As Zn content increases, UTS and YS exhibit a dual-peak trend: initially rising from 1 to 2 at.% Zn, declining at 3 at.% Zn, rebounding at 6 at.% Zn, and finally decreasing at 12 at.% Zn. Peak strength occurs in Mg–2Y–2Zn (UTS: 239 MPa, YS: 130 MPa), while Mg–2Y–1Zn achieves the highest elongation (12.62%).
3. The excellent mechanical properties of Mg–2Y–*x*Zn alloys stem from second-phase strengthening and dynamic phase interactions during plastic deformation. The X-phase serves as the key strengthening component owing to its advantageous crystallographic alignment with the α-Mg matrix and its capacity to generate kink bands. Synergistic strengthening occurs between the X-phase and W-phase, where the W-phase suppresses deformation of the X-phase, thereby enhancing alloy performance. Although both the I-phase and W-phase generate crack initiation sites during deformation, their coexistence triggers a competitive fracture mechanism: the I-phase preferentially fractures to preserve the integrity of the W-phase. The dual mechanisms of synergistic strengthening and competitive fracture between phases offer a novel approach for optimizing the strength–ductility balance in Mg–RE–Zn alloys through phase engineering.

## Figures and Tables

**Table 1:** Chemical compositions of as-cast Mg–2Y–*x*Zn alloys.

Alloy	Nominal Composition	Actual Composition (at.%)		
		Y	Zn	Mg
WZ21	Mg–2Y–1Zn	1.95	0.95	Bal.
WZ22	Mg–2Y–2Zn	1.99	2.03	Bal.
WZ23	Mg–2Y–3Zn	1.92	2.98	Bal.
WZ26	Mg–2Y–6Zn	1.98	6.11	Bal.
WZ212	Mg–2Y–12Zn	1.94	11.83	Bal.

**Table 2:** Solidification sequence order in as-cast Mg–2Y–*x*Zn alloys.

Alloy	Transformation
WZ21	L→α-Mg + L, L→α-Mg + X + Mg_24_Y_5_
WZ22	L→α-Mg + L, L→α-Mg + X + Mg_24_Y_5_ + W
WZ23	L→α-Mg + W
WZ26	L→α-Mg + W, L + W→α-Mg + I
WZ212	L→α-Mg + W, L + W→α-Mg + I

## Data Availability

The original contributions presented in this study are included in the article. Further inquiries can be directed to the corresponding author.
